# A nomogram for predicting upper urinary tract damage risk in children with neurogenic bladder

**DOI:** 10.3389/fped.2022.1050013

**Published:** 2022-12-07

**Authors:** Qi Li, Miao Cai, Qingsong Pu, Shengde Wu, Xing Liu, Tao Lin, Dawei He, Jianguo Wen, Guanghui Wei

**Affiliations:** ^1^ Department of Urology, Children's Hospital of Chongqing Medical University, Chongqing, China; ^2^ Chongqing Key Laboratory of Children Urogenital Development and Tissue Engineering, Ministry of Education Key Laboratory of Child Development and Disorders, National Clinical Research Center for Child Health and Disorders, China International Science and Technology Cooperation Base of Child Development and Critical Disorders, Chongqing Key Laboratory of Pediatrics, Chongqing, China; ^3^ Henan Joint International Pediatric Urodynamic Center, The First Affiliated Hospital of Zhengzhou University, Zhengzhou, China

**Keywords:** child, neurogenic bladder, upper urinary tract damage, nomogram, logistic regression

## Abstract

**Purpose:**

To establish a predictive model for upper urinary tract damage (UUTD) in children with neurogenic bladder (NB) and verify its efficacy.

**Methods:**

A retrospective study was conducted that consisted of a training cohort with 167 NB patients and a validation cohort with 100 NB children. The clinical data of the two groups were compared first, and then univariate and multivariate logistic regression analyses were performed on the training cohort to identify predictors and develop the nomogram. The accuracy and clinical usefulness of the nomogram were verified by receiver operating characteristic (ROC) curve, calibration curve and decision curve analyses.

**Results:**

There were no significant differences in other parameters between the training and validation cohorts except for age (all *P *> 0.05). Recurrent urinary tract infection, bladder compliance, detrusor leak point pressure, overactive bladder and clean intermittent catheterization were identified as predictors and assembled into the nomogram. The nomogram showed good discrimination with the area under the ROC curve (AUC) in the training cohort (0.806, *95% CI*: 0.737–0.874) and validation cohort (0.831, 95% *CI*: 0.753–0.0.909). The calibration curve showed that the nomograms were well calibrated, with no significant difference between the predicted and observed probabilities. Decision curve analysis indicated that the nomogram has good clinical applicability.

**Conclusion:**

This study presents an effective nomogram incorporating five clinical characteristics that can be conveniently applied to assess NB children' risk of progressing to UUTD.

## Introduction

Neurogenic bladder (NB) is a general term for bladder or urethral dysfunction caused by various neuropathic diseases (1). In childhood, the most common cause of NB is myelomeningocele, approximately 50% of children with myelomeningocele have bladder dysfunction (2, 3). The incidence of myelomeningocele in the United States was 1.9 per 10,000 live births (4). Bladder dysfunction in NB patients manifests as detrusor-sphincter dyssynergy, which can lead to incontinence, urinary tract infection (UTI) and vesicoureteral reflux. In advanced stages, renal scarring and renal failure may occur, requiring dialysis or transplantation. Upper urinary tract damage (UUTD) caused by bladder dysfunction is the main cause of death in NB patients (5). The primary goal of NB treatment is to protect renal function, followed by improving bladder function and reducing residual urine volume (5). Therefore, early detection of risk factors for UUTD and taking targeted treatment measures are of great significance for NB children.

Urinary ultrasonography, DMSA, retrograde cystography, and video urodynamics are commonly used to evaluate patients' upper urinary tract function, but these methods have certain limitations. Ultrasound can preliminarily display the morphology and type of renal pelvis, but its results are not accurate enough (6). DMSA can accurately assess renal function, but it will result in radiation exposure (7). Nomogram is a useful and popular visual chart that can predict the probability of an event to guide clinical treatment. At present, there is no predictive nomogram model that can be used to predict the incidence of UUTD in children with NB. In this study, we analysed the clinical data of children with NB and established a simple-to-use nomogram to predict the risk of UUTD. It can help clinicians identify children with high-risk UUTD and take early therapeutic measures to reduce the risk of kidney failure.

## Materials and methods

### Patient selection

The clinical data of 267 NB patients admitted to the Children's Hospital of Chongqing Medical University and the First Affiliated Hospital of Zhengzhou University from January 2011 to June 2022 were retrospectively analysed. The inclusion criteria were as follows: (1) patients diagnosed with NB(5); (2) patients with complete clinical data; and (3) patients aged ≤ 18 years. The exclusion criteria were as follows: patients with other diseases that affect bladder and kidney function (e.g., urethral valve, urethral stricture and nephritis). This study was approved by the Research Ethics Committee of Children's Hospital of Chongqing Medical University (cord number: 2022200).

### Data collection

The 167 NB children from Children's Hospital of Chongqing Medical University were set as the training cohort, and the 100 NB children from the First Affiliated Hospital of Zhengzhou University were set as the validation cohort. The patient's general information, lower urinary tract symptoms, and urodynamic and laboratory examination results were collected from the electronic medical record. General information included age, sex, duration of disease, duration of hospital stay, voiding pattern, history of recurrent UTI, and history of previous spinal surgery. Lower urinary tract symptoms included voiding urgency/frequency, intermittent incontinence, dysuria, and bladder and bowel dysfunction (BBD). Urodynamic results included bladder compliance (BC), detrusor leak point pressure (DLPP) and overactive bladder (OAB). Laboratory examination included urinary ultrasound, retrograde cystography, x-ray of the lumbosacral region and urine culture results. The outcome variable of this research was UUTD. According to the study of Han et al. (8) the standard of UUTD is defined as follows: patients developed vesicoureteral reflux or hydronephrosis. The diagnostic standards of frequency, urgency, dysuria, intermittent urinary incontinence, OAB and DLPP refer to ICCS terms (9). The urodynamic examination procedure was uniformly carried out in accordance with the ICS recommended standards (10).

### Statistical analysis

Statistical analysis was performed using SPSS 20.0 and R 4.0.3 software. Continuous variables conforming to a normal distribution are expressed as *x̄*±*s*. Independent-samples unpaired t tests were used for comparisons between the two groups. Categorical variables are expressed as frequencies and percentages, and the chi-square test was used to compare the differences between the two groups. Univariate logistic regression was performed for all variables in the training cohort. Multivariate logistic regression was performed for variables with *P* < 0.05 in the univariate analysis. The nomogram, receiver operating characteristic (ROC) curve, calibration curve, and decision curve analysis were plotted in R software with relevant packages. A two-sided *α* of less than 0.05 was considered statistically significant.

## Results

### Clinical characteristics

A total of 167 NB children were enrolled in this study, including 149 males and 118 females aged 10.1 ± 3.7 years. The training cohort included 167 children, 97 (58.1%) of them belonged to the UUTD group, whereas 70 (41.9%) children were included in the non-UUTD group. The validation cohort was composed of 100 children, 54 (54.0%) of them belonged to the UUTD. There was no significant difference in other parameters between the training and validation cohorts except for age (all *P *> 0.05) (Table 1). Only CIC patients with recurrent urinary tract infections were treated with antibiotics, other CIC patients were not taking any drugs.

**Table 1:** Demographic and clinical features of patients in the training and validation cohorts.

Characteristics [cases (%)]	All patients (*n* = 267)	Training Cohort (*n* = 167)	Validation Cohort (*n* = 100)	t/x^2^	*P*
**Age (years)**	10.1 ± 3.7	9.6 ± 3.7	11.0 ± 3.7	2.913	0.004
**Gender**				3.356	0.067
Female	118 (44.2%)	81 (48.5%)	37 (37.0%)		
Male	149 (55.8%)	86 (51.5%)	63 (63.0%)		
**Duration of hospital stays (weeks)**				0.283	0.868
< 1	123 (46.1%)	76 (45.5%)	47 (47.0%)		
1–2	47 (17.6%)	31 (18.6%)	16 (16.0%)		
> 2	97 (36.3%)	60 (35.9%)	37 (37.0%)		
**Duration of disease (years)**				3.268	0.195
< 5	138 (51.7%)	92 (55.1%)	46 (46.0%)		
6–10	74 (27.7%)	46 (27.5%)	28 (28.0%)		
> 10	55 (20.6%)	29 (17.4%)	26 (26.0%)		
**Spina bifida**				1.648	0.199
No	104 (39.0%)	70 (41.9%)	34 (34.0%)		
Yes	163 (61.0%)	97 (58.1%)	66 (66.0%)		
**History of spine surgery**				0.015	0.903
No	92 (34.5%)	58 (34.7%)	34 (34.0%)		
Yes	175 (65.5%)	109 (65.3%)	66 (66.0%)		
**Recurrent urinary tract infection**				0.146	0.703
No	87 (32.6%)	53 (31.7%)	34 (34.0%)		
Yes	180 (67.4%)	114 (68.3%)	66 (66.0%)		
**Fever**				2.248	0.134
No	188 (70.4%)	123 (73.7%)	65 (65.0%)		
Yes	79 (29.6%)	44 (26.3%)	35 (35.0%)		
**Voiding Urgency/Frequency**				1.774	0.183
No	193 (72.3%)	116 (69.5%)	77 (77.0%)		
Yes	74 (27.7%)	51 (30.5%)	23 (23.0%)		
**Intermittent incontinence**				0.939	0.333
No	118 (44.2%)	70 (41.9%)	48 (48.0%)		
Yes	149 (55.8%)	97 (58.1%)	52 (52.0%)		
**Dysuria**				1.078	0.299
No	155 (58.1%)	101 (60.5%)	54 (54.0%)		
Yes	112 (41.9%)	66 (39.5%)	46 (46.0%)		
**Bladder and bowel dysfunction**				2.070	0.150
No	138 (51.7%)	92 (55.1%)	46 (46.0%)		
Yes	129 (48.3%)	75 (44.9%)	54 (54.0%)		
Clean intermittent catheterization				0.996	0.318
No	166 (62.2%)	100 (59.9%)	66 (66.0%)		
Yes	101 (37.8%)	67 (40.1%)	34 (34.0%)		
**Bladder compliance (ml/cmH2O)**				0.331	0.565
≥ 20	93 (34.8%)	56 (33.5%)	37 (37.0%)		
< 20	174 (65.2%)	111 (66.5%)	63 (63.0%)		
**Overactive bladder**				0.475	0.491
No	114 (42.7%)	74 (44.3%)	40 (40.0%)		
Yes	153 (57.3%)	93 (55.7%)	60 (60.0%)		
**Detrusor leak point pressure (cmH2O)**				0.815	0.367
< 40	140 (52.4%)	84 (50.3%)	56 (56.0%)		
≥ 40	127 (47.6%)	83 (49.7%)	44 (44.0%)		
**Upper urinary tract damage**				0.425	0.515
No	116 (43.4%)	70 (41.9%)	46 (46.0%)		
Yes	151 (56.6%)	97 (58.1%)	54 (54.0%)		

### Nomogram variable screening

To develop the prediction model, univariate analyses were performed for all variables in 167 children in the training cohort. The results showed that recurrent UTI, DLPP ≥ 40 cmH_2_O, OAB, BC < 20 ml/cmH_2_O and clean intermittent catheterization (CIC) were significantly associated with UUTD (Table 2). Further multivariate logistic regression analysis of the above variables showed that recurrent UTI (*OR *= 2.278, *95% CI*: 1.061∼4.888), DLPP ≥ 40 cmH_2_O (*OR *= 3.588, *95% CI*: 1.703∼7.560), OAB (*OR *= 2.848, *95% CI*: 1.376∼5.894), and BC < 20 ml/cmH_2_O (*OR *= 2.444, *95% CI*: 1.119∼5.341) were risk factors for UUTD, while CIC (*OR *= 0.374, *95% CI*: 0.174∼0.803) was a protective factor (Table 3).

**Table 2:** Univariate analysis of variables associated with upper urinary tract damage in the training cohort.

Characteristics [cases (%)]	non-UUTD group (*n* = 70)	UUTD group (*n* = 97)	t/x^2^	*P*
**Age (years)**	9.9 ± 3.9	9.4 ± 3.5	0.782	0.436
**Gender**			1.538	0.215
Female	30 (42.9%)	51 (52.6%)		
Male	40 (57.1%)	46 (47.4%)		
**Duration of hospital stays (weeks)**			2.206	0.332
< 1	36 (51.4%)	40 (41.2%)		
1–2	10 (14.3%)	21 (21.7%)		
> 2	24 (34.3%)	36 (37.1%)		
**Duration of disease (years)**			3.471	0.176
< 5	43 (61.4%)	49 (50.5%)		
6-10	14 (20.0%)	32 (33.0%)		
> 10	13 (18.6%)	16 (16.5%)		
**Spina bifida**			0.182	0.670
No	28 (40.0%)	42 (43.3%)		
Yes	42 (60.0%)	55 (56.7%)		
**History of spine surgery**			0.187	0.666
No	23 (32.9%)	35 (36.1%)		
Yes	47 (67.1%)	62 (63.9%)		
**Recurrent urinary tract infection**			6.879	0.009
No	30 (42.9%)	23 (23.7%)		
Yes	40 (57.1%)	74 (76.3%)		
**Fever**			3.755	0.053
No	57 (81.4%)	66 (68.0%)		
Yes	13 (18.6%)	31 (32.0%)		
**Voiding Urgency/Frequency**			0.798	0.372
No	46 (65.7%)	70 (72.2%)		
Yes	24 (34.3%)	27 (27.8%)		
**Intermittent incontinence**			0.554	0.457
No	27 (38.6%)	43 (44.3%)		
Yes	43 (61.4%)	54 (55.7%)		
**Dysuria**			3.302	0.069
No	48 (68.6%)	53 (54.6%)		
Yes	22 (31.4%)	44 (45.4%)		
**Bladder and bowel dysfunction**			1.262	0.261
No	35 (50.0%)	57 (58.8%)		
Yes	35 (50.0%)	40 (41.2%)		
**Clean intermittent catheterization**			12.200	<0.001
No	31 (44.3%)	69 (71.1%)		
Yes	39 (55.7%)	28 (28.9%)		
**Bladder compliance (ml/cmH2O)**			14.662	<0.001
≥ 20	35 (50.0%)	21 (21.6%)		
< 20	35 (50.0%)	76 (78.4%)		
**Overactive bladder**			14.310	<0.001
No	43 (61.4%)	31 (32.0%)		
Yes	27 (38.6%)	66 (68.0%)		
**Detrusor leak point pressure (cmH2O)**			9.430	0.002
< 40	45 (64.3%)	39 (40.2%)		
≥ 40	25 (35.7%)	58 (59.8%)		

**Table 3:** Multivariate analysis of variables associated with upper urinary tract damage in the training cohort.

Characteristics	*β*	SE	wald	*P*	OR (95% CI)
**DLPP (cmH2O)**					
< 40					1.000
≥ 40	1.278	0.380	11.293	0.001	3.588 (1.703∼7.560)
**OAB**					
No					1.000
Yes	1.047	0.371	7.956	0.005	2.848 (1.376∼5.894)
**BC (ml/cmH2O)**					
≥ 20					1.000
< 20	0.894	0.399	5.022	0.025	2.444 (1.119∼5.341)
**Recurrent UTI**					
No					1.000
Yes	0.823	0.390	4.461	0.035	2.278 (1.061∼4.888)
**CIC**					
No					1.000
Yes	−0.984	0.390	6.356	0.012	0.374 (0.174∼0.803)

β, regression coefficient; SE, standard error; OR, odds ratio; CI, confidence interval; DLPP, detrusor leak point pressure; OAB, overactive bladder; BC, bladder compliance; UTI, urinary tract infection; CIC, clean intermittent catheterization.

### Nomogram construction and validation

**Figure 1:**
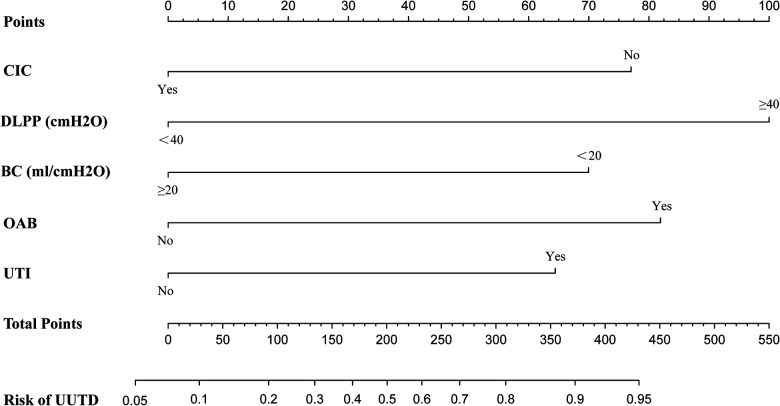
Nomogram to estimate the risk of upper urinary tract damage in patients with neurogenic bladder. CIC, clean intermittent catheterization; DLPP, detrusor leak point pressure; BC, bladder compliance; OAB, overactive bladder; UTI, urinary tract infection; UUTD, upper urinary tract damage.

**Figure 2:**
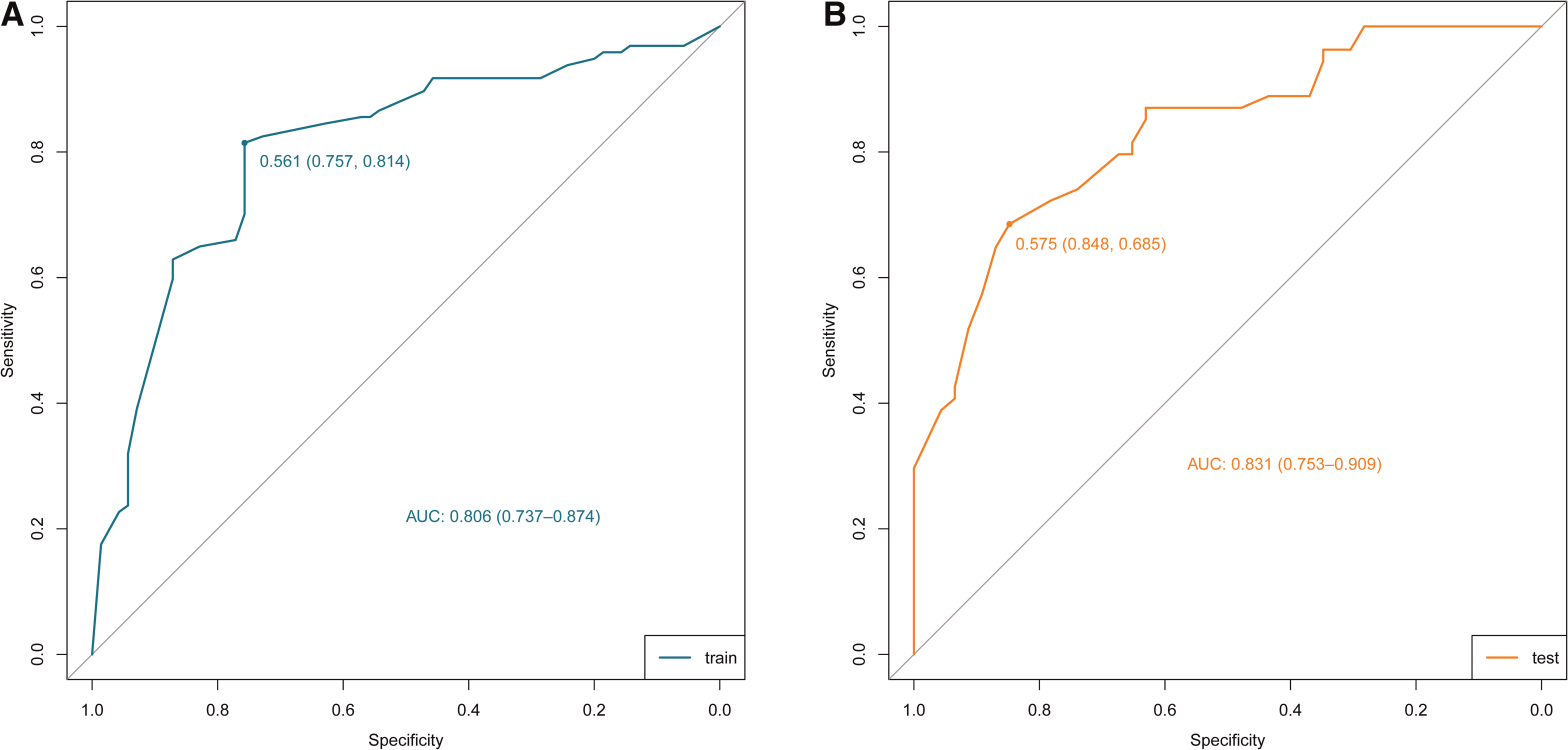
Receiver operating characteristic (ROC) curves of the nomograms in the training and validation cohorts. (**A**) Training cohort. (**B**) Validation cohort.

**Figure 3:**
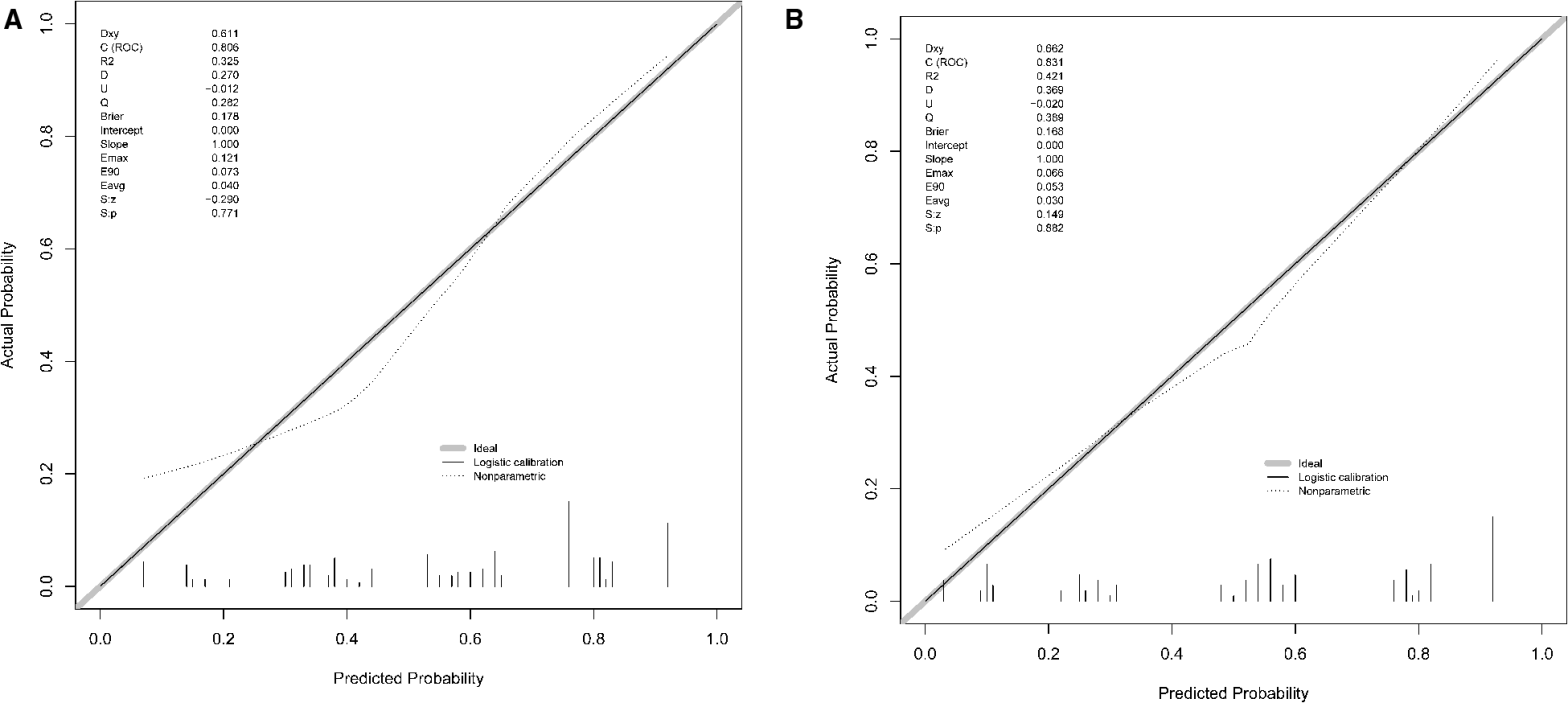
The calibration curve of the nomogram for predicting upper urinary tract damage in the training and testing datasets. (**A**) Training cohort. (**B**) Validation cohort.

**Figure 4:**
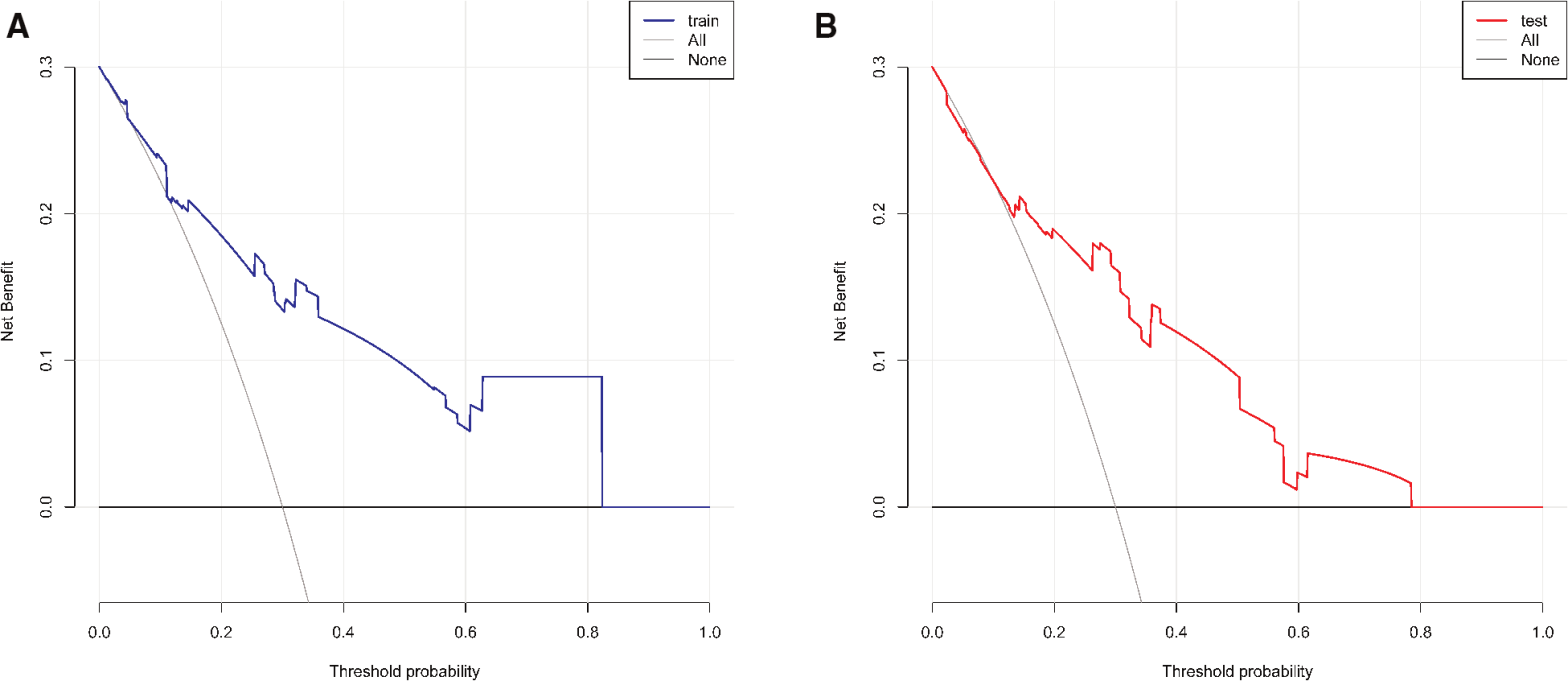
The decision curve analysis for the nomogram. (**A**) Training cohort. (**B**) Validation cohort.

According to the results of multivariate logistic regression analysis, DLPP, OAB, BC, recurrent UTI and CIC were incorporated to establish the prediction model of UUTD, among which DLPP ≥ 40 cmH_2_O was the highest risk factor for the occurrence of UUTD in children with NB (Figure 1). The performance of this nomogram was assessed by area under the ROC curve (AUC), calibration curve and decision curve analysis. The AUC value of the training cohort was 0.806 (*95% CI*: 0.737–0.874), the cut-off value was 56.1%, and the sensitivity and specificity were 0.757 and 0.814, respectively (Figure 2A); the AUC value of the validation cohort was 0.831 (*95% CI*: 0.753–0.909), the cut-off value was 57.5%, and the sensitivity and specificity were 0.848 and 0.685, respectively (Figure 2B). Subsequently, a calibration curve was plotted and evaluated with the unreliability *U* test in the training (*P* = 0.771) and validation cohorts (*P* = 0.882) (Figures 3A,B). The nomograms were well calibrated, with no significant difference between the predicted and the observed probability. To further evaluate the net benefit that patients could receive, decision curve analysis was plotted (Figures 4A,B). As the decision curve analysis indicates, the nomogram model has an obvious net benefit for almost all threshold probabilities, especially in threshold probabilities of 10%–80%.

### Discussion

In this study, we comprehensively evaluated the variables of general information, lower urinary tract symptoms, urodynamic and laboratory results in children with NB and performed univariate and multivariate logistic regression to screen independent risk factors for UUTD. Finally, we established a nomogram model including five factors in the training cohort, which had high sensitivity and specificity to predict the chance of NB in children with UUTD. Moreover, the results of the calibration curve and decision curve analyses indicated that the nomogram had remarkable predictive power and promising benefits for clinical application.

With the progression of the disease, children with NB will experience different degrees of UUTD, which is the major factor that compromises the long-term survival rate (11). Therefore, it is very important to detect UUTD early and take measures to prevent the development of kidney injury. At present, urinary ultrasound, DMSA, retrograde cystography and video urodynamics are commonly used to evaluate upper urinary tract function, but these methods have some limitations. Ultrasound, as a noninvasive examination, can preliminarily display the morphology and type of renal pelvis, but its results are not sufficiently accurate and are greatly influenced by the skill level of the sonographer and subjective factors (6). DMSA can accurately assess renal function in children, but it is expensive and complicated and results in radiation exposure (7). Renal function MRI is a noninvasive, accurate measurements of renal structures and functions for children. It has become increasingly prevalent in research and clinical applications, but its assessment of bladder function is limited (12). The EAU/ESPU recommends that children with NB should undergo video-urodynamic examination as soon as possible to obtain a comprehensive understanding of bladder function and upper urinary tract conditions (5). However, most children are reluctant to perform this examination due to the invasiveness and discomfort of video urodynamics. Therefore, there is still a lack of a convenient and effective method for preliminary assessment of upper urinary tract function in children with NB.

Nomograms, as a pictorial representation of a complex mathematical formula, can use biological and clinical variables to graphically depict a statistical prognostic model and generate a probability of a clinical event for a given individual (13). The physician's professional level varied depending on the hospital level and geographic region; thus, their individual judgments of patients' condition and prognosis were also different. A nomogram provides doctors with a standard calculation formula and reduces diagnostic errors caused by different levels of clinical experience. In addition, help patients in underdeveloped medical resources areas to receive more formal and convenient diagnosis and treatment. Some studies have even shown that nomograms seemed to substantially outperform clinical experts in some respects (14–16).

DLPP was the strongest predictor in this model, and its concept was first proposed in 1,981 by McGuire et al. (17). McGuire et al. followed up 42 children with myelodysplasia for up to 7 years and found that 81% and 68% of the children with DLPP ≥ 40 cmH2O had ureteral dilatation and vesicoureteral reflux, respectively (17). However, none of the children in the DLPP < 40 cmH2O group had vesicoureteral reflux, suggesting that DLPP ≥ 40 cmH2O could be a predictor of UUTD. Similarly, the multivariate logistic regression analysis in this study found that the risk of UUTD in NB children with DLPP ≥ 40 cmH2O was 3.588 times higher than that in NB children with DLPP < 40 cmH2O. However, some researchers believe that using 40 cmH2O as the cut-off value of DLPP to evaluate upper urinary tract function is not reliable. Tarcan et al. assessed upper urinary tract function in 193 children with myelodysplasia and found no significant difference in the number of children with DLPP ≥ 40 cmH2O between the UUTD and non-UUTD groups (18). The reason for this result may be selection bias in the inclusion of the participants. In the study of Tarcan et al., only 70 (36.0%) children had upper urinary tract impairment, which was much lower than 97 (58.1%) in this study. Therefore, taking 40 cmH2O as the cut-off value of DLPP may not be sensitive enough. After they set the cut-off value as DLPP ≥ 20 cmH_2_O, the UUTD could be clearly distinguished (18).

UTI is a common complication in children with NB. The possible reasons for UTI include urinary retention and indwelling catheterization, which can lead to endogenous bacterial colonization in the bladders of NB patients (19). When the bladder interstitial cells are invaded by bacteria, the contraction function of the bladder is inhibited, and the peristaltic function of the ureter is also impaired (20). Once the antireflux mechanisms of the bladder and ureters are disrupted, urine can flow back from the bladder to the ureter or kidney. This increased ureteral/kidney pelvis pressure follows fibrosis and ischemia in the kidney and ultimately develops into kidney failure. Studies have shown that 85% of children with UTI have abnormal DMSA results, and 10.4% of them have permanent renal scarring (21). In this study, the risk of UUTD in children with recurrent UTI was 2.278 times that in children without recurrent UTI. Therefore, attention should be given to UTI in NB children to prevent the occurrence and development of UUTD.

CIC is a new treatment method for detrusor contraction dysfunction in children with NB. It is very effective in reducing bladder pressure, preventing vesicoureteral reflux and improving the prognosis of NB patients (22). The study conducted by Li et al. found that children with early CIC (< 1 year old) have a lower probability of vesicoureteral reflux and UTI than children with late CIC (> 3 years old) (23). Therefore, they believed that early CIC in NB children is important to protect bladder function and prevent kidney damage. However, there is also the view that the children's urinary system has not yet fully developed, the resistance to bacteria is low, and early CIC is likely to cause complications such as UTI (24). The relationship between CIC and UTI seems to be complicated. On the one hand, CIC can prevent UTI by reducing the postvoid residual urine, and on the other hand, improper CIC operation can bring external bacteria into the bladder and cause UTI. Regardless, there is a consensus that earlier CIC is associated with a better prognosis in NB children, which is consistent with the findings of this study. The EAU/ESPU suggests that no medical treatment is needed for asymptomatic bacteriuria caused by CIC, and antibiotics are recommended only when children have recurrent UTI or fever (5).

### Limitations

The study had some limitations. First, the number of samples used to build the model is limited, and more sample sizes are needed to improve the model. Second, this retrospective study may have selection bias, and further prospective validation is needed to evaluate its applicability. Third, NB children may present with urethral dysfunction, and urethral pressure was not evaluated in this study. Nevertheless, this study established the first UUTD risk model based on various clinical parameters in children with NB. This model can help clinicians make a preliminary assessment of upper urinary tract function in children with NB and has high accuracy and clinical applicability.

## Conclusion

A prognostic model based on five risk factors for predicting the risk of UUTD in NB children was built and demonstrated good discrimination and clinical applicability. It helps clinicians identify children with high-risk UUTD and take early therapeutic measures to reduce the risk of kidney failure.

## Data Availability

The raw data supporting the conclusions of this article will be made available by the authors, without undue reservation.
